# Detection of Amyloid Plaques Targeted by Bifunctional USPIO in Alzheimer’s Disease Transgenic Mice Using Magnetic Resonance Microimaging

**DOI:** 10.1371/journal.pone.0057097

**Published:** 2013-02-27

**Authors:** Youssef Zaim Wadghiri, Jialin Li, Jinhuan Wang, Dung Minh Hoang, Yanjie Sun, Hong Xu, Wai Tsui, Yongsheng Li, Allal Boutajangout, Andrew Wang, Mony de Leon, Thomas Wisniewski

**Affiliations:** 1 Department of Radiology, New York University School of Medicine, New York, New York, United States of America; 2 Department of Neurology, New York University School of Medicine, New York, New York, United States of America; 3 Department of Psychiatry, New York University School of Medicine, New York, New York, United States of America; 4 Department of Pathology, New York University School of Medicine, New York, New York, United States of America; 5 Nathan Kline Institute, Orangeburg, New York, United States of America; 6 Ocean NanoTech, LLC, Springdale, Arkansas, United States of America; 7 Tianjin Huanhu Hospital, Tianjin, China; Graduate School of Pharmaceutical Sciences, The University of Tokyo, Japan

## Abstract

Amyloid plaques are a key pathological hallmark of Alzheimer’s disease (AD). The detection of amyloid plaques in the brain is important for the diagnosis of AD, as well as for following potential amyloid targeting therapeutic interventions. Our group has developed several contrast agents to detect amyloid plaques *in vivo* using magnetic resonance microimaging (µMRI) in AD transgenic mice, where we used mannitol to enhance blood brain barrier (BBB) permeability. In the present study, we used bifunctional ultrasmall superparamagnetic iron oxide (USPIO) nanoparticles, chemically coupled with Aβ1-42 peptide to image amyloid plaque deposition in the mouse brain. We coupled the nanoparticles to polyethylene glycol (PEG) in order to improve BBB permeability. These USPIO-PEG-Aβ1-42 nanoparticles were injected intravenously in AD model transgenic mice followed by initial *in vivo* and subsequent *ex vivo* μMRI. A 3D gradient multi-echo sequence was used for imaging with a 100 µm isotropic resolution. The amyloid plaques detected by T2*-weighted μMRI were confirmed with matched histological sections. The region of interest-based quantitative measurement of T2* values obtained from the *in vivo* μMRI showed contrast injected AD Tg mice had significantly reduced T2* values compared to wild-type mice. In addition, the *ex vivo* scans were examined with voxel-based analysis (VBA) using statistical parametric mapping (SPM) for comparison of USPIO-PEG-Aβ1-42 injected AD transgenic and USPIO alone injected AD transgenic mice. The regional differences seen by VBA in the USPIO-PEG-Aβ1-42 injected AD transgenic correlated with the amyloid plaque distribution histologically. Our results indicate that USPIO-PEG-Aβ1-42 can be used for amyloid plaque detection *in vivo* by intravenous injection without the need to co-inject an agent which increases permeability of the BBB. This technique could aid the development of novel amyloid targeting drugs by allowing therapeutic effects to be followed longitudinally in model AD mice.

## Introduction

The extracellular accumulations of amyloid β (Aβ) peptides as plaques and cerebral amyloid angiopathy (CAA), as well as intracellular neurofibrillary tangles (NFTs) are pathological hallmarks of Alzheimer’s disease (AD) [1]. AD is currently the 6^th^ commonest cause of death in the USA, with the numbers of affected individuals expected to rise significantly globally [2]. The definitive diagnosis of AD is still based on histological confirmation of these pathological features at postmortem examination. With improvements in imaging technology in recent years, there has been a growing interest in developing methods to visualize Aβ plaques *in vivo* in mice, using methods that could eventually be applied to humans [3]–[5]. PET is currently being used in humans to visualize amyloid deposits in AD, mild cognitively impaired and normal aged individuals [6]–[8]. However, the low spatial resolution of PET does not allow the visualization of individual plaques and it is not clear if it will be able to detect the earliest stages of amyloid deposition. The current therapeutic approaches under development are likely to be most effective when initiated at very early stages of amyloid deposition [9]–[12]; hence early detection is a critical issue. MRI has a much higher spatial resolution than PET and is also superior to optical imaging for assessment of the whole brain. In addition MRI is widely available for animal and patient imaging and does not require the injection of a radiotracer. In prior studies we demonstrated the first *in vivo* observation of Aβ plaques in AD Tg mice using intra-carotid (ic) injections, with mannitol to increase permeability of the BBB, with Aβ1-40 peptide tagged with either gadolinium diethylenetriaminepentaacetic acid (Gd-DPTA) or monocrystalline iron oxide nanoparticles (MION) [13], [14]. We were also able to visualize *in vivo* amyloid deposits without the use of amyloid ligands based on the T2 and T2* relaxation time induced by iron bound in plaques and/or the dense structure of the plaques themselves [15]–[19]. Our studies corroborated reports by others using post-mortem AD brain tissue [20] and AD transgenic mouse brain tissue (*ex vivo* [21]–[23] and *in vivo* [22], [24], [25]). However, the limitation of this approach is that only the most “mature” plaques and the small subset of plaques with a diameter >50μm can be visualized when using relatively short acquisition times (<2 hours) to ensure the possibility of *in vivo* imaging; hence, under these conditions only a small percentage of amyloid deposits can be detected directly and early amyloid plaques can’t be visualized using this method. We subsequently reported that Aβ-Gd-DPTA can be used to follow a therapeutic amyloid reducing effect [26]. We also tried to increase the BBB permeability of the ligand by incorporation of polylysine residues (K6) on Aβ, with the use of K6-Aβ-Gd-DPTA [18]. This improved plaque visualization; however, we still needed to inject the ligand by ic injection with mannitol to allow passage across the BBB. A limitation in the use of Aβ-Gd-DPTA is a very short plasma ½ life of ∼3 minutes. Therefore we turned to stronger magnetic labels such as ultrasmall superparamagnetic iron oxide nanoparticles (USPIO) to overcome the limitations of requiring ic injections (which are very invasive) and mannitol co-injection [13], [19], [27]. USPIO are known to have much longer plasma ½ life of 24–36hr in humans [28], [29] and ∼2 hours in mice [30]. Furthermore the strong darkening effects exhibited by these particles on T2 and T2* imaging sequences gives a greater contrast to noise ratio and allows a smaller dose of ligand to be used. However, this technique still requires femoral co-injection of mannitol for CNS imaging as USPIO do not cross the intact BBB. The attachment of polyethylene glycol (PEG) to the surface of USPIO has been shown to inhibit its uptake by the reticuloendothelial system and prolongs circulation, thus promoting delivery to the CNS [29], [31], [32]. Furthermore the PEGylation of nanoparticles has been reported to allow penetration of the intact BBB to some extent [33]–[35]. Hence in the current study we have produced bi-functional USPIO particles coupled to both Aβ1-42 and PEG, in order to assess their utility to visualize amyloid plaques *in vivo* using AD model transgenic mice following intravenous injection, without the co-injection of an agent to increase permeability of the BBB.

## Materials and Methods

### Ethics Statement

Animal studies were approved by the NYU School of Medicine Institutional Animal Care and Use Committee (protocol 100609-03) and were consistent with the recommendations of the American Veterinary Association.

### Animals

12 to 18-month old APP/PS1 transgenic mice, and age-matched wild-type (C57Bl/6J) control mice were used in this study (Table 1). This transgenic line is based on the overexpression of both amyloid precursor protein mutations (APP_K670N/M671L_) and mutant presenilin-1 (PS1_M146L_) [36]. The genotype of each mouse was confirmed by PCR analysis using samples of mouse-tail DNA.

**Table 1:** Summary of μMRI Scans.

Mice	Set 1: *In vivo* scans with USPIO-PEG-Aβ1-42	Set 2: *In vivo* scans with USPIO-Aβ1-42	Set 3: *In vivo* scans with USPIO alone	Set 4: *ex vivo* scans with USPIO-PEG-Aβ1-42	Set 5: *ex vivo* scans with USPIO-Aβ1-42	Set 6: *ex vivo* scans with USPIO alone
APP/PS1 Tg	12	9	9	12 (from set 1)	9 (from set 2)	9 (from set 3)
Wild-type	12			12 (from set 1)		

Shows a summary of the µMRI scans performed in each set of mice.

### Contrast agents

Aβ 1-42 peptide was synthesized in the W.M. Keck Facility at Yale University (New Haven, CT). Details of synthesis, purification, and sequence verification were described previously [27], [37]. The ultrasmall superparamagnetic iron oxide (USPIO) nanoparticles (10 mgFe/ml, Ocean Nanotech) were linked to Aβ1-42 and PEG using standard EDC/NHS, as previously described [27]. Aβ peptides are known to have high affinity binding to each other, in particular monomeric Aβ to deposited, aggregated Aβ; hence, coating USPIO nanoparticles with monomeric Aβ1-42 is expected to target the particles to amyloid plaques, as demonstrated in prior publications [13], [18], [19], [27], [38]. After coupling, the free Aβ peptide was separated from the nanoparticles by centrifugation at 25,000rpm for 25 min. This produced a clear supernatant and a brownish pellet. The supernatant was taken for protein assay. We verified that chemical coupling was ∼90% efficient with ∼1 mg of Aβ peptides coupling to 1 mg of USPIO particles using the BCA protein assay. The pellet was washed several times with PBS and an aliquot was run on 16.5% SDS-PAGE under nonreducing conditions, followed by immunoblotting using anti-Aβ antibodies (6E10; Covance Inc., Princeton, NJ) to verify the coupling of the peptide to the USPIO. Briefly, samples were separated by SDS-PAGE and transferred to nitrocellulose membranes. After blocking with 5% milk, the membrane was incubated with monoclonal anti-Aβ antibody 6E10 (1∶2000), and subsequently with horseradish peroxidase-conjugated goat anti-mouse secondary antibody (1∶5000, Thermo Scientific, Rockford, IL). The signal was detected by the enhanced chemiluminescence (ECL) Western blotting detection reagents (Amersham Biosciences, Piscataway, NJ).

### Binding affinity and toxicity studies

Experiments were performed to assess the ability of USPIO-PEG-Aβ1-42 to bind to Aβ1-40 which an abundant peptide in AD amyloid deposits. The interaction between USPIO-PEG-Aβ1-42 and Aβ1-40 was evaluated by enzyme-linked immunosorbent solid phase assays (ELISA), as described previously [27]. Briefly, freshly dissolved Aβ1-40 (synthesized by the W.M. Keck Facility at Yale University) was coated overnight onto polystyrene microtiter plates and then the plates were blocked with superblock. Increasing concentrations of USPIO-PEG-Aβ1-42 in Tris-buffered saline were applied to Aβ1-40 coated wells for 3h. Bound USPIO-PEG-Aβ1-42 was detected with an Aβ1-42 specific antibody (R422) that does not cross react with Aβ1-40 [26]. All binding studies were performed in triplicate. After washing, the plates were incubated for 1 hr with an anti-rabbit horseradish peroxidase-linked antibody (Amersham Life Science), developed for 15 min with a TMB peroxidase kit (Bio-Rad, Hercules, CA), and quantified at 450 nm on a a Spectramax M2 microplate reader (Molecular Devices, Sunnyvale, CA). The data were analyzed by a nonlinear regression fit algorithm in Prism5.0 (GraphPad Software, San Diego, CA). Negative controls for nonspecific binding included wells without coating Aβ1-40 and omission of the anti-Aβ1-42 or secondary antibodies, as well as wells incubated with non-coupled USPIO.

The potential neurotoxicity of USPIO-PEG-Aβ1-42 was assessed in a human neuroblastoma cell line (SK-N-SH) using the 3-(4,5-dimethylthiazol-2-yl)-5-(3-carboxymethoxyphenyl)-2-(4-sulfophenyl)-2H-tetrazolium (MTS) Cell Proliferation Assay (Promega, Madison, WI) and compared to the known toxicity of Aβ1-42 [39], [40], using methods we have previously described [18], [27], [40,40]. 10,000 cells per 100μl of culture medium per well were plated onto flat-bottom, 96 -well microtiter plates and allowed to attach overnight and were treated with USPIO-PEG-Aβ1-42, Aβ1-42 alone and uncoupled USPIO for 72 hours. The Aβ1-42 alone and uncoupled USPIO were positive and negative controls, respectively. The dose of each peptide was 10 µM. This concentration of Aβ1-42 has been shown to be toxic in this tissue culture model [40]. The concentration of USPIO particles in the media of the cells treated with USPIO alone was matched to the cells treated with USPIO-PEG-Aβ1-42. Subsequently, the MTS colorimetric solution was added and allowed to incubate at 37 °C for 2-3 hours. MTS was bio-reduced by cells into a formazan product that was soluble in tissue culture medium. The absorbance of the formazan at 490 nm was measured directly from 96-well plates in a Spectramax M2 and using SoftMaxPro software Version 4.8. Cell viability were determined as percent of control, with control being non-treated cells. The statistical significance of USPIO-PEG-Aβ1-42, Aβ1-42 alone and uncoupled USPIO toxicity was analyzed by one way ANOVA followed by a Neuman-Keuls post-hoc test (GraphPad Prism, version 5; GraphPad Inc., San Diego, CA, USA).

### Mouse injections

USPIO-PEG-Aβ1–42, USPIO- Aβ1–42 or USPIO alone was dissolved in 300 µl of 0.9% sodium chloride (pH 7.4) at a dose of 0.2 mmol Fe/kg body weight immediately before infusion. The USPIO were infused via the right femoral vein using a PHD2000 computer-controlled syringe pump (Harvard Apparatus, Hollison, MA) at a rate of 60 µl/min., as we previously described [18], [27]. Anesthesia was induced with 2.5% isoflurane in air for 3–5 minutes, followed by 1–1.5% isoflurane in air to maintain anesthesia.

### *In vivo* MRI brain imaging

All mice were scanned 4hs after intravenous injection of the contrast agent to ensure maximum plaque detection with minimal nonspecific labeling of blood vessels (see Table 1 for summary of mouse groups). The four hour post ligand injection imaging time was based on optimization we performed in earlier studies [13], [18], [19]. The control group injected with uncoupled USPIO particles alone (n = 9), were equivalent to mice imaged without a contrast ligand, since USPIO particles do not cross the BBB. For *in vivo* imaging the mice were anesthetized with 2.5% isoflurane in 75% NO_2_ plus 22% O_2_. For maintenance of anesthesia the isoflurane was reduced to 1–1.5%. Body temperature of the mice was maintained between 35–37°C using a warm-water blanket. All MRI scans were performed on a 7T micro-MRI system consisting of a 7-Telsa 200-mm horizontal bore magnet (Magnex Scientific, UK) equipped with an actively shielded gradient coil (Bruker BGA-9S; ID 90 mm, 750 mT/m gradient strength, 100 s rise time) interfaced to a Bruker Biospec Avance 2 console.

For *in vivo* μMRI a modified 3D spoiled gradient recalled multi-echo (SPGRME) sequence was used to acquire an additional self-gated signal on the readout dephasing gradient within each TR [41]. The gating signal was used retrospectively to generate artifact free four-echo image reconstruction sets with the following parameters: 100 µm isotropic spatial resolution; repetition time (TR)  =  80 ms; bandwidth (BW)  =  75 kHz, matrix  = 256×256×128; first echo time (TE)  =  4.1 ms with echo spacing (ES)  =  4.1 ms (effective subsequent echo times are as follow: 8.2, 12.3, 16.4 ms), flip angle (FA)  =  20^o^ and imaging time  =  2 hr 20 min. The advantage of the 3D-imaging approach is that the image set can be reprocessed in any desired slice orientation using Analyze software (AnalyzeDirect, Overland Park, KS), facilitating image comparison during co-registration with histology. Among the four-echo train image sets acquired, the third echo was used to generate a T2*-weighted (TE = 12.3 ms) image subset for both plaque visualization and VBA.

The apparent transverse relaxation time T2* was measured using the four-echo train image sets described above in several brain regions defined by region of interest (ROI). ROIs were manually drawn at the level of the hippocampus and cortex with NIH imageJ software (imagej.nih.gov/ij). The cortical ROIs were defined as dorsomedial from the cingulate cortex and extended ventrolaterally to the rhinal fissure within the left or right hemisphere. Fourteen ROIs in the cortex and eight ROIs in the hippocampus were measured per animal. A ROI in the cerebellum was used as an internal control. The mean signal intensity of all voxels contained within each ROI was subsequently used for fitting. The four-point decay curves obtained from the echo-train dataset were fit to a single exponential using a non linear least square algorithm with Origin 7.5 (OriginLab Corp., Northampton, MA) to obtain the T2* measurements.

### *Ex vivo* MRI brain imaging

Animals were anesthetized with sodium pentobarbital (150 mg/kg, i.p.) and perfused transaortically with 0.1 M PBS, pH 7.4, followed by 4% paraformaldehyde, and their brains were extracted for *ex vivo* MRI and histology. For *ex vivo* imaging, a dedicated apparatus was designed to scan up to eight brains simultaneously overnight using a homemade coil circularly polarized dual Litzcage (ID  =  33 mm, length 52 mm) to fit a 60 cm^3^ syringe. Individual brains were glued into place in each quadrant of the syringe plunger and immersed in Fomblin (Solvay Solexis Inc., Thorofare, NJ). Fomblin provided a completely dark background around the brains being imaged [26]. The imaging sequence was a 3D multi-gradient echo (MGE) sequence with 100-µm isotropic spatial resolution, TR  =  50 ms, bandwidth (BW)  =  1001kHz, matrix  =  256×256×512, TE  =  4.07 ms, ES  =  6.7 ms (effective subsequent echo times are as follow: 10.8, 17.5, 24.2 ms), FA  =  20°, imaging time  =  8 h 11 min.

### Histological Studies

After *ex vivo* MRI, the brains were removed from Fomblin and placed in 2% DMSO/20% glycerol in PBS overnight or until sectioning. Serial coronal sections (40 µm) were cut and every fifth section were stained with a combination of 6E10 (epitope Aβ3-8) and 4G8 (epitope Aβ17-24), both monoclonal anti-Aβ antibodies (Covance, Emeryville, CA), as previously described [13], [16], [18], [26]. A combination of two anti-Aβ monoclonal antibodies, with different epitopes, is used to ensure all amyloid deposits are immunolabeled, as well as to reduce the possibility that USPIO-PEG-Aβ1-42 binding to plaques would mask the deposited Aβ epitopes and prevent immunodetection [27], [42], [43]. Briefly, free floating sections were incubated in 6E10/4G8 antibodies at a dilution of 1∶1000 for 2 h. An immunodetection kit (MOM; Vector Laboratories, Burlingame, CA) was used with the anti-mouse IgG secondary antibody reacted for 1 hr at a 1∶1000 dilution. Antibody staining was revealed with 3,3′-diaminobenzidine (DAB; Sigma-Aldrich, St. Louis, MO) with nickel ammonium sulfate intensification.

Some sections were double stained for Aβ deposits using a red detection substrate and for iron (to allow visualization of the USPIO particles) using a Perl stain which gives a blue color. Sections were first immunolabeled with 6E10/4G8 antibodies followed by use of a red alkaline phosphatase substrate kit (Vector Laboratories) and then Perl’s staining. Briefly, free floating sections were incubated with 6E10/4G8 antibodies at a dilution of 1∶1000 for 2 hours, followed by application of alkaline phosphatase labeled horse anti-mouse IgG secondary antibodies for 1 hour at a 1:200 dilution. The sections were then mounted on slides, left to dry and Perl’s iron stain was performed. Briefly, slide-mounted sections were placed in a solution containing 10% potassium ferrocyanide and 20% hydrochloric acid for 1 hours under sunlight. The sections were then washed in distilled water and cover slipped with Aqua Poly/Mount (Polysciences, Inc).

### Voxel-based Analysis (VBA)

VBA analysis was performed using MATLAB 9 (the MathWorks, Natick, MA) and SPM5 (Wellcome Trust Center for Neuroimaging, University College London, UK) with SPMMouse toolbox [44]. The steps involved in processing data were similar to that of human data processing and as we published previously [27], [45]. Briefly, the acquired *ex vivo* mouse brain images were spatially normalized to the control C57Bl/6J mouse brain template in the SPMMouse toolbox and segmented into grey matter probabilistic density maps. These maps were then smoothed with a 300 µm isotropic Gaussian kernel to correct for imperfect registration and make the distribution of data more Gaussian. Two-tailed *t*-tests were then performed on these tissue maps to identify group-wise changes in grey matter structure in the framework of the general linear model (GLM).

After injection of the USPIO-PEG-Aβ1-42, amyloid plaques were visualized on MRI as hypointense spots characterized by loss of signal intensity, as we have observed following USPIO- Aβ1-42 injections with mannitol [27]. Regional specific differences were assessed statistically using the GLM/univariate analysis with a one-tailed *T* statistics, showing voxels of lower intensity in APP/PS1 Tg mice compared to wild-type mice. We considered *p* <0.05 (uncorrected for multiple comparisons) to indicate statistical significance for individual voxels, with a minimum cluster size of 500 voxels as previously reported for rodent VBA analysis[46], [47].

## Results

### Analysis of Aβ1-42 coupled USPIO

**Figure 1:**
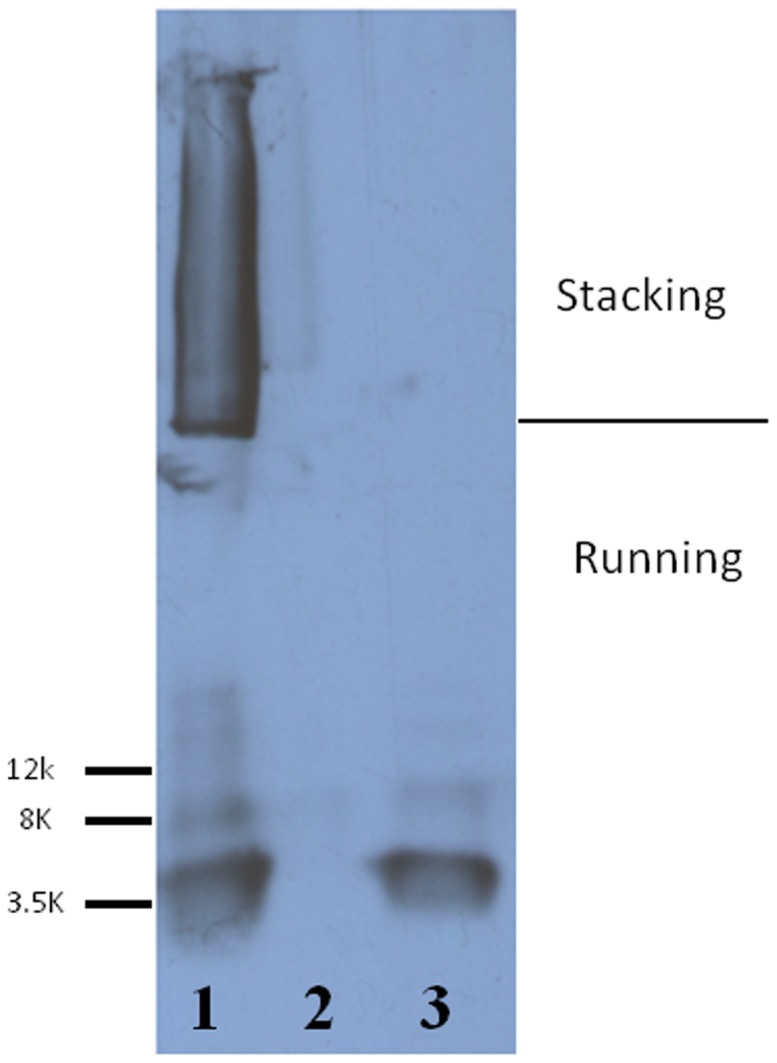
Immunoblotting showed the coupling of Aβ1-42 to USPIO using anti-Aβ monoclonal antibody 6E10. Lane 1 shows USPIO-PEG-Aβ1-42 in which Aβ1-42 coupled to nanoparticles is mainly in the stacking gel. A small proportion of Aβ1-42 which was not covalently bound to the particles entered the running gel. Lane 2 shows USPIO alone as negative control, with no bands seen. Lane 3 shows synthetic Aβ1-42 as a positive control, where the two bands seen are monomeric and dimeric Aβ1-42.

The coupling of Aβ1-42 to USPIO-PEG was evaluated by immunoblotting using monoclonal anti-Aβ 6E10 antibody (Covance Inc., Princeton, NJ). The majority of the USPIO-PEG-Aβ1-42 did not transfer onto the nitrocellulose membrane (see Figure 1). Of the proportion of the USPIO-PEG-Aβ1-42 that was transferred, the majority of the beads stayed in the stacking gel; a small portion of the Aβ1-42 that was not covalently bound to the beads entered the running gel and can be seen as 4.5, 9.0 and higher kDa bands, corresponding to monomeric, dimeric and aggregated Aβ1-42. This portion of Aβ1-42 was presumably bound to the beads by hydrophobic interactions, which is how we previously had bound Aβ1-40 to MION particles [13]. USPIO beads without coupled Aβ1-42 were run in lane 2 and no bands were seen. Synthetic Aβ1-42 peptide alone was run in lane 3 with two bands being seen, corresponding to monomeric and dimeric Aβ1-42. These results were similar to what we previously reported with USPIO-Aβ1-42 particles [27].

**Figure 2:**
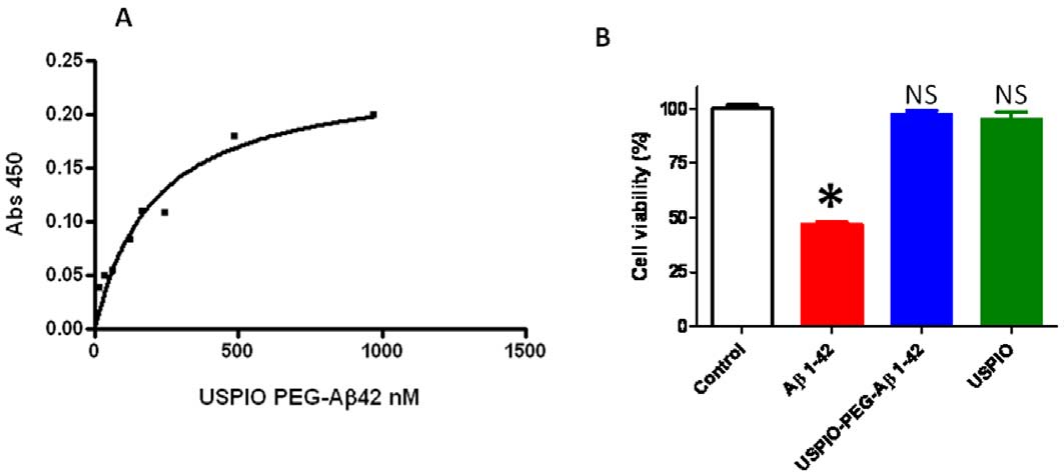
A shows the binding between USPIO-PEG-Aβ1-42 and Aβ1-40 assessed by ELISA. The K_D_ of USPIO-PEG-Aβ1-42 to Aβ1-40 binding was calculated to be 206.7nM±42.09. B shows a bar graph illustrating tissue culture toxicity studies using USPIO-PEG-Aβ1-42, Aβ1-42 alone and uncoupled USPIO on N2a cells (mean ± s.d.). Cell viability of untreated control cells is set at 100%. There was no evidence of significant toxicity of USPIO-PEG-Aβ1-42 at a concentration of 10 µM as compared to Aβ1-42 alone at the same 10 µM concentration, under these conditions. No toxicity of USPIO particles alone was evident. One way ANOVA analysis gave a p<0.0001 for a difference between the groups. Newman-Keuls post-hoc analysis showed Aβ1-42 versus control, Aβ1-42 versus USPIO-PEG-Aβ1-42 and Aβ1-42 versus USPIO to be significantly different (p<0.001, p<0.001, p<0.001, respectively). USPIO alone versus control untreated cells, USPIO-PEG-Aβ1-42 versus control untreated cells and USPIO versus USPIO-PEG-Aβ1-42 did not differ significantly. NS: not significant.

The binding affinity between Aβ and USPIO-Aβ1-42 was assessed by ELISA. The K_D_ of USPIO-PEG-Aβ1-42 to Aβ1-40 was calculated to be 206.7nM±42.09 (see Figure 2A), which was slightly lower affinity than our previously reported binding of USPIO-Aβ1-42 to Aβ1-40 [27], suggesting that the addition of PEG slightly interfered with the interaction of USPIO-PEG-Aβ1-42 to free Aβ1-40. In our toxicity studies we found that Aβ1-42 alone at 10μM was toxic, as expected, producing a 50% reduction in cell viability (Figure 2B). One way ANOVA analysis gave a p<0.0001 for a difference between the groups. Newman-Keuls post-hoc analysis showed Aβ1-42 versus control, Aβ1-42 versus USPIO-PEG-Aβ1-42 and Aβ1-42 versus USPIO to be significantly different (p<0.001, p<0.001, p<0.001, respectively). USPIO versus control untreated cells, USPIO-Aβ1-42 versus control untreated cells and USPIO versus USPIO-PEG-Aβ1-42 did not differ significantly. Hence there was no evidence of significant toxicity of bound Aβ1-42 on the USPIO particles at a concentration of 10μM as compared to Aβ1-42 alone at the same concentration, under these conditions. As shown in Figure 1, a small percentage of Aβ1-42 is bound by hydrophobic interactions with USPIO and could potentially become detached. There was no evidence of toxicity related to this potential problem in our tissue culture toxicity assay or *in vivo* in the mice.

### µMRI studies

**Figure 3:**
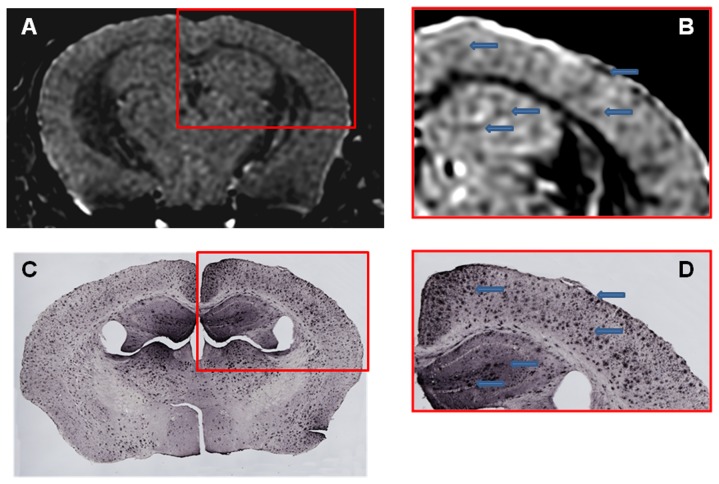
Amyloid plaques were detected with *in vivo* µMRI after intravenous femoral injection of USPIO-PEG-Aβ1-42. *In vivo* T2*-weighted μMRI images show a 14 month-old APP/PS1 Tg mouse. **B** shows a higher magnification of the area in the red box of shown in **A**. C shows the matching tissue section to **A** immunolabeled with anti-Aβ 4G8/6E10. **D** shows a higher magnification of the area in the red box of **C**. Arrowhead highlight some of the matching dark spots seen by μMRI with the immunolabeled tissue sections.

**Figure 4:**
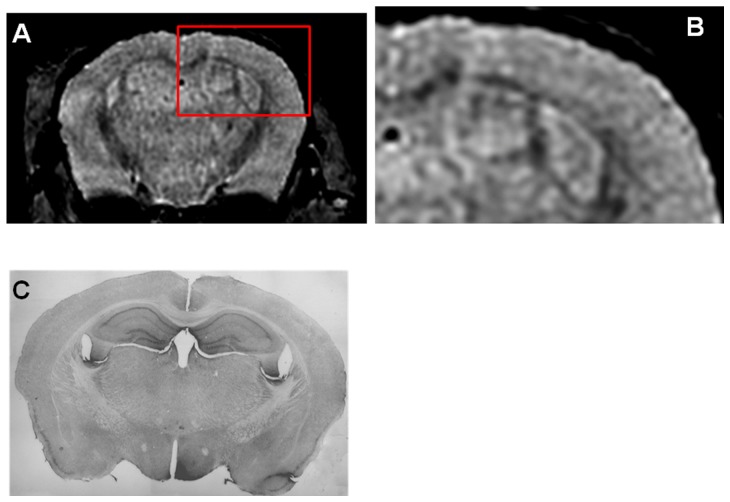
Shows an *in vivo* μMRI of a 16 month old wild-type mouse after intravenous femoral injection of USPIO-PEG-Aβ1-42. **B** shows a higher magnification of the area in the red box of shown in **A**. Some dark spots are also evident in A (likely corresponding to blood vessels) but far fewer than seen in Figure **3A** or Figure **4A**. C shows the matching tissue section to **A** immunolabeled with anti-Aβ 4G8/6E10. No amyloid deposits are evident, as expected.

Femoral injection of USPIO-PEG-Aβ1-42, resulted in the detection of numerous dark spots in APP/PS1 mice using *in vivo* (N = 12) T2*-based gradient echo (Third echo, TE  =  12.3 ms) µMRI (Figure 3). The patterns of hypointense spots were similar to the Aβ distribution seen in matched histological sections (see arrowheads in Figure 3). However, due to difference in the slice thickness and slice orientation between the μMRI images (100 μm) and the tissue sections (40 μm) it is not possible to match exactly all dark spots see by μMRI and the immunohistochemistry. In control wild-type mice (with no amyloid deposits) following USPIO-PEG-Aβ1-42 some dark spots are also seen (Figure 4); however, these are far fewer than in the Tg mice. These likely correspond to blood vessels. In APP/PS1 mice injected with USPIO alone a few dark spots are also seen, some of these correspond to large amyloid plaques which can be visualized without injection of an amyloid binding ligand. The USPIO alone injected APP/PS1 mice are the equivalent of mice imaged without injection of any amyloid binding ligand, since USPIO particles (without being coupled to PEG) do not cross an undamaged BBB [27], [29], [48]. As we have previously published, under the imaging conditions we are using, only a very small percentage of amyloid lesions can be detected without the injection of an amyloid binding ligand [15]–[19].

**Figure 5:**
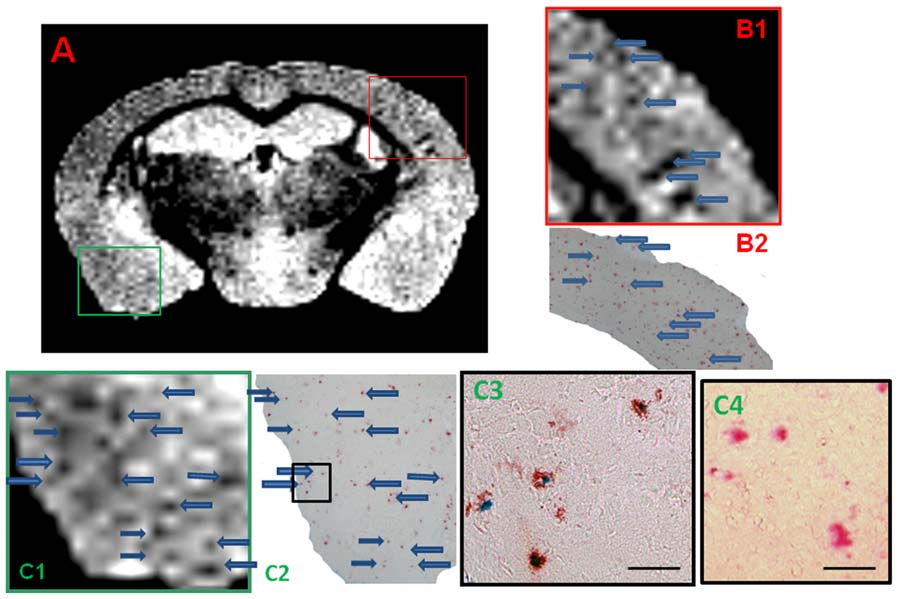
Shows an *ex vivo* μMRI coronal cut at the level of the anterior hippocampus of a 15 month-old APP/PS1 Tg injected with USPIO-PEG-Aβ1-42, showing numerous dark spots. In B1 a higher magnification of the μMRI in the red box in **A** is shown. This is matched to amyloid plaque immunolabeling (in red) shown in **B2**. Arrows highlight some matches between the μMRI and histology. Figure **C1** is higher magnification of the μMRI in the green box in **A**. This is matched to double amyloid plaque immunolabeling and Perl iron staining in **C2** and **C3**. Arrows highlight some of the matches between the μMRI in **C1** and immunolabeling in **C2**. **C3** is a higher magnification of the black box in **C2**. **C4** shows and example of double amyloid plaque immunolabeling and Perl iron staining in an APP/PS1 mouse injected with USPIO alone. Only the red immunolabeling of amyloid plaques is seen with no co-label of iron. Co-labeling of amyloid plaques with anti-Aβ 4G8/6E10 (in red) and Perl iron stain (in blue) highlights the co-deposition of USPIO-PEG-Aβ1-42 on amyloid deposits in **C2** and **C3** (scale bar  =  100 microns).

**Figure 6:**
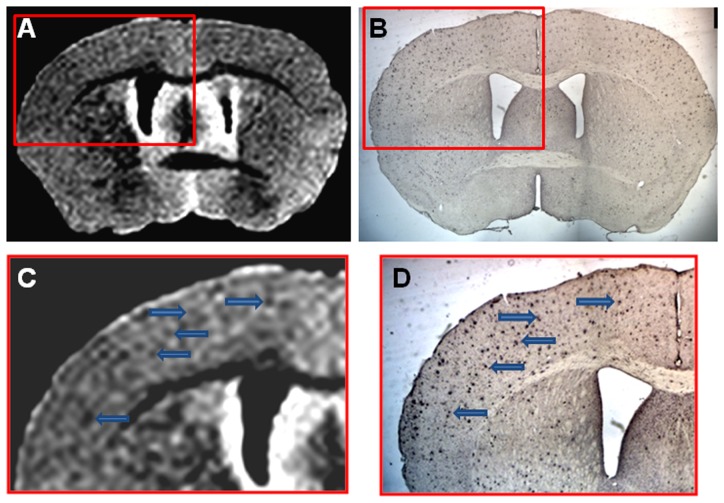
Shows an example of *ex vivo* μMRI, at the level of the area cinguli of the cerebral cortex, of a 14 month-old APP/PS1 Tg injected with USPIO-PEG-Aβ1-42, showing numerous dark spots. This is matched to a tissue section immunolabeled with anti-Aβ 4G8/6E10 in B. **C** is higher magnification of the μMRI in the red box in **A**, while **D** is a higher magnification of the tissue section immunolabeling seen in **B**.

Amyloid plaques were also detected in USPIO-PEG-Aβ1-42 injected transgenic mice on *ex vivo* T2*-weighted μMRI (Third echo, TE  =  17.5) extracted from the four-echo dataset (Figure 5). Figure 5A shows a coronal section of an APP/PS1 Tg mouse with many dark spots. Figure 5B shows a higher magnification of the area within the red box of Figure 5A. Figure 5B1 shows the μMRI, while 5B2 showed the corresponding amyloid plaque immunolabeling on a tissue section. Figure 5C shows a higher magnification of the area within the green box of Figure 5A. Figure 5C1 shows the μMRI, while 5C2 showed the corresponding amyloid plaque immunolabeling on a tissue section. Figure 5C3 shows a higher magnification of the area within the black of 5C2. Amyloid plaques are seen in red (using the red alkaline phosphatase substrate) while iron deposition is seen in blue (using Perl staining). The iron labeling indicates co-deposition of USPIO particles on the amyloid plaques. The iron labeling we performed only stained extracellular amyloid deposition. There was no evidence of USPIO-PEG-Aβ1-42 uptake into microglia. However, to more fully address whether microglia around plaques take up some of the bound USPIO-PEG-Aβ1-42, electron microscopic studies would need to be done. Perl staining alone of amyloid plaques in these APP/PS1 Tg mice injected with USPIO alone did not give any blue staining, under the conditions used (Figure 5C4). In Figure 6 a further example is shown in an APP/PS1 Tg mouse injected with USPIO-PEG-Aβ1-42 of the matching of amyloid plaques detected by μMRI to tissue immunolabeling of Aβ deposits.

### T2* measurement

In order to compare the different groups of *in vivo* imaged mice using unbiased methods the absolute T2* measurements in the cortex, hippocampus and cerebellum were compared (see Table 2). Lower T2* measurements corresponded to more dark spots and greater plaque burden. APP/PS1 mice injected with USPIO-PEG-Aβ1-42 showed a significant decrease in T2* value in both the cortex and hippocampus, compared with wild-type mice (Table 2) injected with USPIO-PEG-Aβ1-42 (p<0.00001) comparing measurements from the cortex and hippocampus, but there were no differences in T2* comparing the cerebellum where there are no amyloid deposits. The cerebellum is an area of the brain not subject to amyloid deposition in this mouse model [12], [36]. Similarly the APP/PS1 mice injected with USPIO-PEG-Aβ1-42 differed significantly from APP/PS1 mice injected with USPIO alone (as USPIO alone does not cross the BBB) and APP/PS1 mice injected with USPIO-Aβ1-42 (as USPIO-Aβ1-42 without PEG has very limited BBB passage), when comparing the cortex and hippocampus. There was no significant difference in T2* in these groups of mice when comparing the cerebellum. This data indicates that sufficient USPIO-PEG-Aβ1-42 is able to cross the BBB *in vivo* and result in T2* changes in USPIO-PEG-Aβ1-42 injected APP/PS1 mice which can be attributed to specific binding to Aβ plaques, given the significantly lower T2* measurements in areas of the brain affected with Aβ deposition (cortex and hippocampus). This contrasts with the lack of differences among the different mouse groups in the *in vivo* T2* measurements in the cerebellum, where there is no amyloid deposition.

**Table 2:** T2* Measurements.

Mouse Groups	Left Cortex	Right Cortex	Left Hippocampus	Right Hippocampus	Cerebellum
APP/PS1 Tg from Set 1	38.63±1.35	38.62±0.97	39.84±0.54	39.88±0.61	43.5±2.19
APP/PS1 Tg from Set 2	40.2±1.5	40.26±1.69	42.44±2.98	42.07±2.8	43.14±2.16
APP/PS1 Tg from set 3	44.6±1.08	42.32±1.58	44.57±2.5	45.22±2.64	44.97±2.51
Wild-type from set 1	42.2±0.99	41.97±1.02	42.2±0.99	43.23±1.47	43.5±3.65
p-values of Tg from set 1 compared to Tg from set 2	0.02156	0.01095	0.007489	0.01558	0.71402
p-values of Tg from set 1 compared to Tg from set 3	<0.00001	<0.00001	<0.00001	<0.00001	0.92865
p-values of Tg compared to WT from set 1	<0.00001	<0.00001	<0.00001	<0.00001	0.99863

Shows a comparison of the absolute T2* values in the different mouse groups and the p-values comparing different groups using a two-tailed Students t-test. This data indicates that sufficient USPIO-PEG-Aβ1-42 is able to cross the BBB *in vivo* and result in T2* changes in USPIO-PEG-Aβ1-42 injected APP/PS1 mice, which can be attributed to specific binding to Aβ plaques, given the significantly lower T2* measurements in areas of the brain affected with Aβ deposition (cortex and hippocampus).

### VBA using SPM

**Figure 7:**
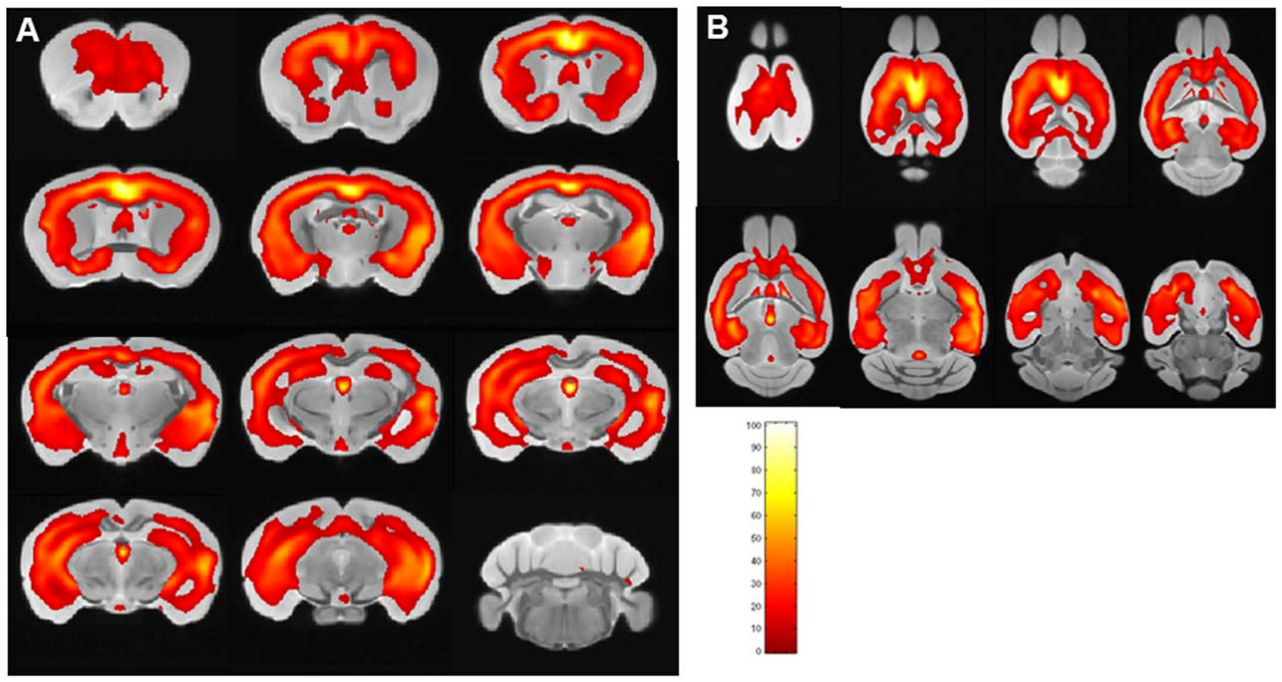
Shows areas of the brain are shown which differed by voxel-based analysis of *ex vivo* μMRI comparing USPIO-PEG-Aβ1-42 injected APP/PS1 Tg mice (n = 12) and APP/PS1 Tg mice (n = 12) injected with USPIO alone (*p*<0.01). The regions of differences mirror the distribution of amyloid plaques histologically, being mainly in the cortex and hippocampus. Color bar units are the T-score. **A** shows areas which are significantly different in coronal sections, while **B** shows areas which are significantly different in horizontal sections.

VPA using SPM was used to compare the different groups of mice. Figure 7 shows (in both coronal [7A] and horizontal views [7B]) the comparison of APP/PS1 mice injected with USPIO-PEG-Aβ1-42 (n = 12) compared to APP/PS1 mice injected with USPIO alone (n = 9, USPIO alone does not cross the BBB). A marked difference between the APP/PS1 mice injected with USPIO alone versus APP/PS1 mice injected with USPIO-PEG-Aβ1-42 is evident. Clusters of voxels representing statistically significant darker areas of the brain corresponding to amyloid deposits (p<0.01). The largest significantly different clusters were found in the cortex and hippocampus. The regional distribution of differences between the two groups mirrors the distribution of amyloid plaques histologically. No differences were seen in the cerebellum where there are no plaques.

**Figure 8:**
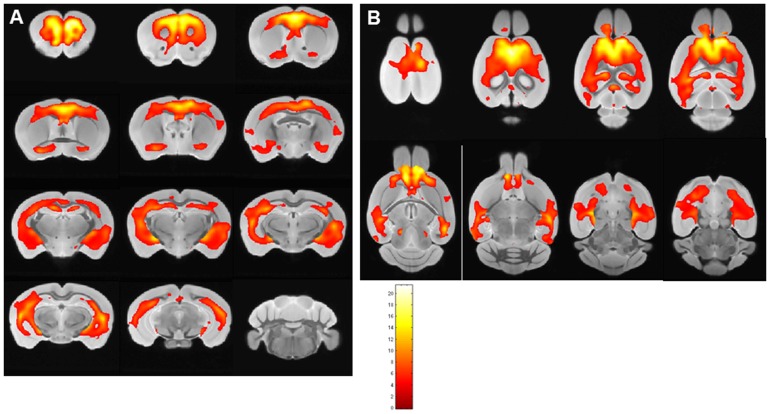
Shows areas of the brain are shown which differed by voxel-based analysis of *ex vivo* μMRI comparing USPIO-PEG-Aβ1-42 injected APP/PS1 Tg mice (n = 12) and wild-type mice (n = 12) injected with USPIO-PEG-Aβ1-42 (*p*<0.05). The regions of differences mirror the distribution of amyloid plaques histologically, being mainly in the cortex and hippocampus. Color bar units are the T-score. **A** shows areas which are significantly different in coronal sections, while **B** shows areas which are significantly different in horizontal sections.

A similar analysis was done in Figure 8 comparing APP/PS1 mice (n = 12 to wild-type mice (n = 12), both subjected to USPIO-PEG-Aβ1-42 injection (p<0.05). Again the largest significantly different clusters were found in the cortex and hippocampus. No differences were seen in the cerebellum where there are no plaques.

## Discussion

There is great interest in the development of better methods to detect AD related lesions early in the disease process and to be able to follow changes in lesion load as the disease progresses, as well as having the ability to follow changes related to therapeutic interventions. Our studies report the ability of bi-functional USPIO coupled to both PEG and Aβ1-42 to cross the BBB and target amyloid deposits following intravenous femoral injections. The PEGylation of nanoparticles has been previously demonstrated to allow penetration of the intact BBB to some extent [33]–[35], while our own prior studies have shown that Aβ1-42 or Aβ homologous peptides can target iron nanoparticles or gadolinium to plaques, provided the BBB can be overcome [13], [18], [19], [27]. In our prior studies we have used an intra-carotid or an intravenous route with a requirement for co-injections of mannitol to disrupt the BBB [13], [19], [26], [27]. Although this is a well studied and effective means of increasing the permeability of the BBB, allowing the CNS delivery of various agents such as neurotrophic factors, viral vectors and USPIO [49]–[51], it has a short duration of action (∼15 min in rodents) [50] and it is associated with some toxicity. Our use of USPIO-PEG-Aβ1-42 to image amyloid plaques, without any other agent to induce specific BBB permeation is therefore more suitable for longitudinal studies in AD model animals and has greater potential to be used in patients under research settings. The plasma 1/2 life of USPIO has previously been shown to be ∼ 2 hr in mice [52], while in humans the plasma ½ life is 24-36hr [28], [29]. This greater circulation time enhances the ability of USPIO-PEG-Aβ1-42 to cross the BBB and eventually to label brain plaques. The binding affinity (K_D_) of our USPIO-PEG-Aβ1-42 to the Aβ peptide was ∼206 nM, representing relatively high affinity binding. The dosage we used of USPIO-PEG-Aβ1-42 at 0.2 mmol/kg body weight is in the range of clinical use. For MRI applications contrast agents are typically injected intravenously at ∼ 0.1 mmol/kg for diagnostic purposes [19], [27]. Another cause of potential concern with USPIO-PEG-Aβ1-42 is the known toxicity of Aβ1-42 at micromolar concentrations [39]. However, Aβ peptide toxicity is greatly dependent on its conformational/aggregation state as well as its concentration [53]. We report that under our conditions where the Aβ1-42 is coupled to USPIO-PEG there is no evidence of toxicity in a standard tissue culture model and there was also no evidence of toxicity in our experimental animals.

We show that the USPIO-PEG-Aβ1-42 is able to cross the BBB and label plaques by direct comparison to immunolabeling of tissue sections (Figures 3 and 5). However, due to difference in the slice thickness and angulation of the μMRI images (100 μm) and the tissue sections (40 µm) it is not possible to co-register all dark spots seen on the μMRI images. Furthermore in wild-type animals with no amyloid plaques, some dark spots were also evident in some mice with no ligand injection (data not shown) or after USPIO-PEG-Aβ1-42 injections (Figure 4). Many of these dark spots were related to blood vessels, as previously described [15], [19], [27]. This false positive identification can be ruled out manually through careful three-dimensional analysis of contiguous slices. In addition slight retention of USPIO nanoparticles in normal healthy mice following injection has also been observed [15], [27]. Hence it is important to verify specific labeling of amyloid plaques using unbiased methodologies. In our *in vivo* μMRI we used absolute T2* measurements to show that in APP/PS1 Tg mice injected with USPIO-PEG-Aβ1-42, areas of the brain with amyloid deposits (cortex and hippocampus) were significant lower (corresponding to more dark spots) compared to wild-type mice injected with USPIO-PEG-Aβ1-42, as well as, APP/PS1 Tg mice injected with either UPSIO-Aβ1-42 or USPIO alone (see Table 2). Importantly, T2* measurements in these 4 sets of mice did not differ significantly in the cerebellum, where there is no amyloid deposition. VBA analysis was used to compare the different *ex vivo* imaged mouse groups. We could not use T2* measurements to compare the post-mortem imaging, as fixation altered the T2* values markedly. VBA analysis showed that areas of the brain with extensive amyloid deposits differed significantly in APP/PS1 Tg mice injected with USPIO-PEG-Aβ1-42, compared to APP/PS1 Tg mice injected with either UPSIO-Aβ1-42 or USPIO alone (Figure 7), as well as to wild-type mice injected with USPIO-PEG-Aβ1-42 (Figure 8). Areas of the brain without amyloid deposits such as the cerebellum did not differ in these groups of mice by VBA analysis, suggesting the specificity of the USPIO-PEG-Aβ1-42 labeling of amyloid plaques.

These findings support the use of PEGylated UPSIO coupled to a targeting peptide which binds to Aβ as a method for the specific detection of AD plaques using μMRI, in AD model mice without the use of any agent to increase the permeability of the BBB. Such a method of plaque detection will be helpful in the development and evaluation of potential novel therapeutic interventions to reduce amyloid deposits. Several USPIO contrast agents have been approved for clinical use for the imaging of abdominal viscera [29]. Studies of our bifunctional USPIO particles for the imaging of AD amyloid lesions are on-going in non-human primates, prior to any potential future human testing.
